# Influence of UV light irradiation on the corrosion behavior of electrodeposited Ni and Cu nanocrystalline foils

**DOI:** 10.1038/s41598-020-59420-6

**Published:** 2020-02-20

**Authors:** Shu-hao Deng, Hao Lu, D. Y. Li

**Affiliations:** 1 School of Materials Science and Engineering, Central South University, Changsha, 410083 China; 2 Department of Chemical and Materials Engineering, University of Alberta, Edmonton, Alberta T6G 1H9 Canada; 3 The Key Laboratory of Nonferrous Metal Materials Science and Engineering, ministry of education, Changsha, 410083 China

**Keywords:** Structural materials, Electrochemistry

## Abstract

Influence of ultraviolet (UV) light irradiation on the corrosion behavior of electrodeposited Ni and Cu nanocrystalline foils in 3.5% NaCl solution was studied by means of electrochemical methods, electron work function (EWF) analysis, and characterization with atomic force microscopy (AFM) and X-ray photoelectron spectroscopy (XPS). It was demonstrated that the influence of solar light on corrosion of the metals was non-negligible, which could be very different for different materials. The UV light irradiation resulted in an increase in corrosion resistance of the Cu foil but showed an opposite influence on that of the Ni foil. Based on surface state analysis, it was concluded that the UV irradiation altered the surface oxide films. The UV light induced the formation of Cu_2_O on Cu, which is more stable and compacted than naturally formed CuO film. However, the UV light accelerated the formation of Ni_2_O_3,_ which is loose, porous and brittle, compared to naturally formed NiO on Ni. The changes in oxide films were responsible for the opposite variations in the corrosion behavior of the Cu and Ni nanocrystalline foils caused by the UV light irradiation.

## Introduction

Electrodeposited metal foils have been widely used in many industrial applications because of their desired properties, easy fabrication and low cost. For example, Cu and Ni foils are used in electromagnetic shielding, electronics (Printed Circuit Board, PCB), current-collector of battery, package, anode and many other fields^1–6^. Because of their wide range of applications, considerable research has been conducted to investigate Cu, Ni and their alloys. For realistic applications, it is vital to know the stability of Cu and Ni in different environments and corresponding mechanisms. The frequent corrosion mode of Cu and Ni is atmospheric corrosion, which has been broadly investigated^7–14^. Many studies on corrosion of Cu and Ni in NaCl solutions have also been carried out, since NaCl is one of important aggressive media, which has impact on corrosion of metals especially in ocean environment^9,10^. NaCl can deteriorate formed oxide films on copper and nickel^9^ under nature condition, especially at high relative humidity (RH) levels, thus accelerating degradation of the metals.

Although the corrosion behaviors of Cu and Ni in atmospheric and marine environments have been widely investigated, the materials are usually used under solar light irradiation or illumination. In particular, the UV light with higher radiant energy in the solar light spectrum (wavelength from 200 nm to 650 nm, corresponding to the radiant energy from 6.2 eV to 1.9 eV) could result in unexpected changes in their corrosion behavior, which is however much less known. Such effect would be stronger if the materials are in the nanocrystalline state because the high-density grain boundaries raise the energy state and promote atomic diffusion along grain boundaries. Although limited studies were conducted on corrosion of steel, Cu, Zn, and Al alloys under UV irradiation^15–29^, results of the studies are not always consistent. Besides, there is almost no investigation reported in the literature regarding the influence of light irradiation on corrosion of nickel, an important base metal having a wide range of industrial applications. Regarding the inconsistency in the reported studies, very different effects of UV light on corrosion of Cu and Cu alloys were reported. It is reported that the UV light can promote corrosion of Cu^24,27,28^. Graedel T. E’S, *et al*.^28^ report that the UV light increases Cu sulfidation by a factor of 1.5 to 2 in the presence of H_2_S, resulting in decreased resistance to corrosion. While Hidalgo’s study suggests that UV light has no effect on the corrosion rate of Cu^29^, and some other studies show that the UV light increases the anti-corrosion property of Cu or Cu alloy due to the formation of more stable passive films^25,26^. Thus, more investigates are needed in order to have the discrepancy clarified.

Nanocrystalline electrodeposits have attracted continuous interests due to the fact that the nanocrystalline structure considerably changes properties with increased strength, wear resistance and corrosion resistance^30–35^. The high-density grain boundaries strongly influence the surface activity and oxidation behavior. It is thus of interest to see how the nanocrystalline metals respond to corrosive environments under UA light irradiation. However, there are almost no such studies reported in the literature. These motivate the study on how the UV light could influence the performances of nanocrystalline Cu and Ni in corrosive environments and what are underlying mechanisms.

Objectives of this study are to 1) evaluate respective effects of UV light irradiation on the corrosion behaviors of nanocrystalline Cu and Ni foils in a 3.5%NaCl solution, and 2) clarify the mechanisms for different effects of UA light on corrosion behaviors of the Cu and Ni foils through investigating the UV irradiation – induced variations in oxide films formed on the Cu and Ni foils.

## Experimental Details

Cu and Ni foils were prepared by electrodeposition using established procedures reported in the literature^4,36^. The purity of the Cu and Ni foils was 99.9% and their dimensions were 30 × 30 × 0.015 mm and 30 × 30 × 0.030 mm, respectively.

In order to evaluate the influence of UV light irradiation on the corrosion behavior, a 6 W UV lamp (UVP 95-0007-06 Model) with 254 nm wavelength (corresponding to the radiant energy of 4.84 eV) was used as a light source. Prior to experiments, each sample was ultrasonically cleaned for 5 minutes in acetone of analytical grade and then dried. To evaluate their corrosion behavior under the effect of UV light, the samples were immersed in a 3.5% NaCl solution at the ambient temperature of 20 °C under irradiation of light with its wavelength equal to 254 nm for 8 hours per day; and the critical time when the first hole appeared on the sample resulted in corrosion was recorded. For comparison, another group of samples were tested under the same condition but in dark. This corrosion assessment was performed with ten repeated experiments, five of them were carried out in the dark and another five performed under the irradiation condition.

In order to obtain more information on the effect of UV irradiation on the corrosion behavior of the foils, electrochemical experiments were performed to provide additional information on how the UV irradiation affects the surface activity in the ambient condition, which helps understand the effect of UV irradiation on the proneness of the foils to corrosion. Unlike the immersion test, the electrochemical experiment is very fast, while the effect of UV light irradiation on corrosion process is slow. It is difficult to feasibly evaluate the effect of UV light irradiation on electrochemical behavior of the foils in the NaCl solution. Thus, the experiment was arranged in the following way. A IM6Ex potentiostat was employed to assess the compactness of the product layer on Ni and Cu surfaces and the corrosion resistances of the nanocrystalline Ni and Cu foils before and after successive exposure to the UV light having its radiant energy equal to 4.84 eV for 36 hours. The exposure time was determined based on the Ni immersion corrosion result, which showed that the UV light exposure time was sufficient to result in detectable response in terms of corrosion in the ambient environment (20 °C, humidity of 65%). For the tests carried out in the 3.5% NaCl solution, the foil was the research electrode, Hg/Hg_2_Cl_2_ saturated with KCl was the comparison electrode, and a Pt foil was used as the counter electrode.

A MIRA3 LMH model scanning electron microscope (SEM, TESCAN) and a multimode 8 atomic force microscopy (AFM, Bruker) were used to observe the surface morphology and corrosion products on the nickel and copper foils. The scan size of AFM for the observation was set as 5μm × 5μm for Cu and 850 nm × 850 nm for Ni. The microstructure constituents of the foils were analyzed with D/max-2500 X-ray diffraction (XRD) within a 2θ range of 20~100° and the scanning rate was 8°·s^−1^. The grain size of the foils was also estimated from the XRD analysis, which was consistent with direct AFM observations.

Electron work functions (EWF) of the Ni and Cu foils before and after UV light irradiation were measured using a Kelvin probe (KP technology, UK). Since the EWF experiment is also fast, all the samples were also irradiated by the 254 nm UV light (corresponding to the radiant energy of 4.84 eV) for 36 hours before the EWF was measured. Adhesion and conductance of foil surfaces before and after UV light irradiation were also investigated using the multimode 8 atomic force microscope (AFM) at 20 °C with the humidity of 65%. In order to determine the oxide films formed with and without UV irradiation, the oxide films were characterized with Kratos AXIS 165 X-ray photoelectron spectroscopy.

## Results and Discussion

### Characterization

**Figure 1:**
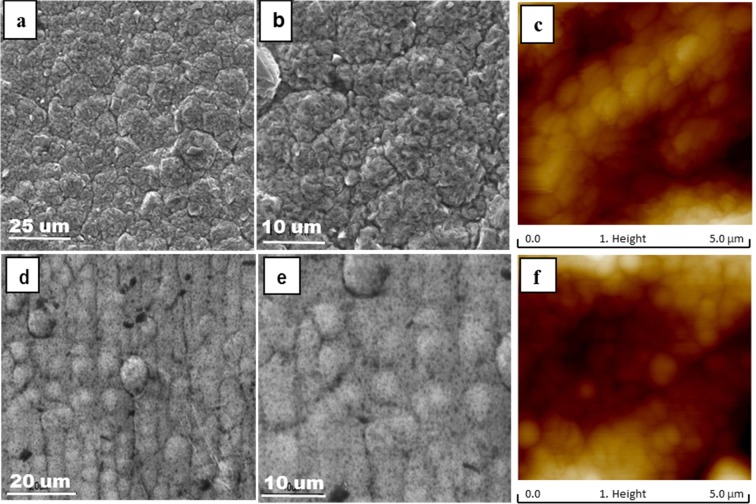
SEM and AFM images of Cu and Ni foils at different scales. (**a,b**) SEM of Cu, (**c**) AFM of Cu, (**d,e**) SEM of Ni, and (**f**) AFM of Ni.

Morphologies of the electrodeposited Ni and Cu foils were examined and are illustrated in Fig. 1, in which micrographs of the Cu foil are presented in figures (a–c), and those of the Ni foil are shown in figures (d–f), including SEM (black and white) and AFM (colored).

In Fig. 1, one may see that the electrodeposited Cu and Ni are uniform, fine-grained and dense because of high cathode polarization. No micro cracks are visible, suggesting that the internal stress could be low and few defects exist in the deposits. The Cu foil appears to be compact with a granular or multi- angular structure. There are some sharp arris on the Cu foil but the AFM image with a closer view only shows a uniform spherical structure. Based on the AFM image, the grain size of the electrodeposited Cu is about 70~80 nm.

Under SEM, the Ni foil also shows spherical nodule morphology, and the spherical nodules consist of smaller spherical nodules. The AFM image shows that the Ni foil has a uniform spherical structure and its grain size is around 20~30 nm, smaller than that of Cu.

**Figure 2:**
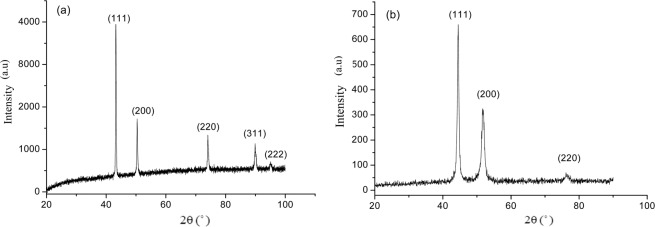
XRD patterns of the fabricated Cu and Ni foils: (**a**) Cu, and (**b**) Ni.

Figure 2(a,b) illustrate XRD patterns of the Cu and Ni foils. In Fig. 2(a), the peaks at 43.4°, 50.4°, 74.1°, 89.9° and 95.2° come from face-centered cubic Cu planes, (111), (200), (220), (311) and (222), respectively, which is in agreement with JCPDS 04-0836. The grain size of Cu foil is 62 nm, calculated using the Scherrer formula, which is consistent with that obtained from the AFM observation.

In Fig. 2(b), the peaks at 44.3°, 51.7°, and 76.2° come from face-centered cubic Ni planes, (111), (200) and (220), respectively, which match the information given in JCPDS 65-2865. The grain size of Ni foil is 26.5 nm, calculated using the Scherrer formula, which is consistent with that observed in the AFM analysis.

The XRD patterns show that both the electrodeposited Cu and Ni foils are (111) preferred textured. Studies show that (111) textured Ni and Cu foils possess higher corrosion resistance^8,9,13,37^, which is understandable since the (111) plane has the lowest surface energy, corresponding to the highest surface stability.

The grain sizes of both the Cu and Ni foils are smaller than 100 nm, which should have marked influence the corrosion behavior through affecting the formation of oxide scale and its properties. As mentioned earlier, the high-density grain boundaries in the nanocrystalline substrates promote atomic diffusion, helping the development of oxide film with reduced defects at the oxide/substrate interface, thus improving the oxide integrity and adherence to the substrate.

### Corrosion

#### The corrosion behavior

Table 1 presents results of immersion tests (in the 3.5% NaCl solution) for the Cu and Ni foils under UV light irradiation and in dark, respectively. The purpose of the designed tests was to ascertain the critical period of time at which corrosion took place on the samples with and without the UV light irradiation, respectively. As demonstrated, the UV light affected the corrosion behaviors of Cu and Ni differently. The UV light increased the anti-corrosion property of the Cu foil, but significantly deteriorated the Ni foil. Though Ni foil demonstrated better corrosion resistance than Cu foil in the natural condition, the corrosion resistance of Cu was better than that of Ni when UV light irradiation was involved.

**Table 1:** Results of the immersion tests (in 3.5% NaCl) with and without (in dark) UV irradiation for evaluating corrosion resistance, evaluated by the time prior to corrosion occurrence (pitting), of the Cu and Ni foils.

Material	Cu		Ni	
	UV light	In dark	UV light	In dark
Time prior to corrosion	12 days	7 days	6 days	48 days

**Figure 3:**
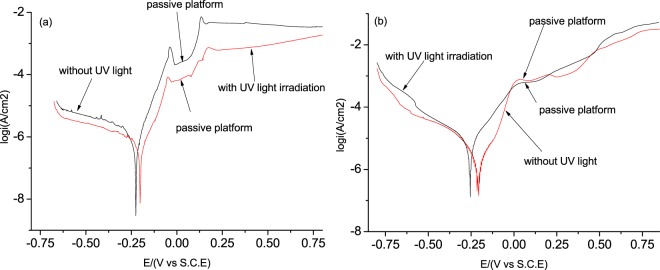
Polarization curves of (**a**) Cu and (**b**) Ni foils in a 3.5% NaCl solution.

Table 2 and Fig. 3 present results of the electrochemical tests and recorded polarization curves for the Cu and Ni foils before and after exposure to UV light irradiation, respectively. The results are consistent with those obtained from the immersion tests (Table 1) i.e. the corrosion resistance of Cu was improved by the UV light irradiation, while that of Ni was deteriorated by the UV light irradiation.

**Table 2:** Results of the electrochemical tests for Cu and Ni foils in 3.5% NaCl solution before and after Exposure to UV light irradiation, respectively.

Material	Cu		Ni	
	without	with UV light	without	with UV light
E_corr_/V	−0.225	−0.202	−0.144	−0.21
I_corr_/(A·cm^−2^)	5.26 × 10^−6^	1.63 × 10^−6^	2.53 × 10^−6^	8.42 × 10^−6^

In Fig. 3(a,b), the shapes of the polarization curves before and after UV light irradiation are similar. The corrosion potential of Cu is shifted positively after exposure to the UV light with reduced corrosion current density, and the passivation platform of polarization curve is broadened. These indicate that the oxidation product on the Cu foil increased the anti-corrosion property of the Cu foil after exposure to the UV light. However, the UV irradiation resulted in an opposite effect on the properties of the Ni foil, indicating that the UV light has a negative impact on passivation of Ni and accelerates corrosion of the Ni foil.

**Figure 4:**
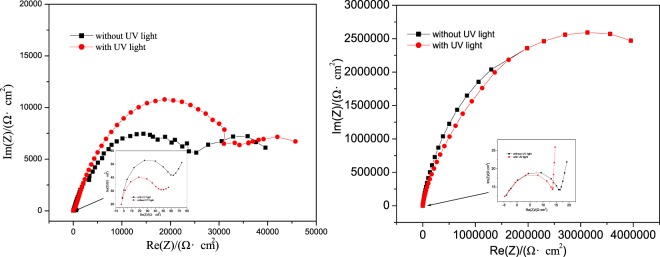
Electrochemical impedance spectra of (**a**) Cu and (**b**) Ni foils in the 3.5% NaCl solution at open circuit potential (OCP).

Electrochemical impedance spectrum (EIS) is often employed to study the metal surface state, and it can also be used to estimate the protectiveness of a corrosion product scale formed on the metal surface. Figure 4 and Table 3 show impedance spectra of open circuit potential (OCP) and the extracted information obtained by the EIS of the Cu and Ni, respectively. The Nyquist spectra are similar, beginning with a tiny circle followed by a big semicircle. One may see from the results that the rate controlled step of metal corrosion is a charge-transfer process. The first small circle (from 1 MHz~10 KHz) demonstrates the resistance of corrosion media passing through the oxidation film on the metal surface, and the value of the resistance (R_1_) represents the density of the oxidation film. The followed larger semicircle reflects the metal corrosion process, and the value of the resistance (R_2_) shows the corrosion resistance of bare substrate in the corrosion medium.

**Table 3:** Information obtained from the EIS of Cu and Ni foils in 3.5% NaCl solution at open circuit potential(OCP).

Material	Cu		Ni	
	without	with UV light	without	with UV light
R_1_/(Ω·cm^2^)	50	64.5	20.5	18.5
R_2_/(Ω·cm^2^)	1.5 × 10^4^	2.0 × 10^4^	3.5 × 10^6^	3.5 × 10^6^

Comparing the initial tiny circles before and after the UV light irradiation, it can be seen that the resistance of the oxide film on Cu surface to both corrosion and the charge-transfer increased after the exposure to the UV light irradiation. Such increases imply that the oxide film formed on the Cu foil is relatively dense and improves the anti-corrosion property of the Cu foil after exposed to UV light illumination. However, the resistance of the oxide film on Ni to corrosion decreased after exposure to the UV light irradiation, and the charge-transfer resistance did not change much. Such decreases suggest that the oxide film formed on the Ni foil, which was exposed to UV light irradiation, is relatively loose and discontinuous with lower coverage on the substrate. Such an incomplete passive film cannot prevent the penetration of electrolyte. The looser oxide film also results in local corrosion battery and thus decreases the corrosion resistance of the metal (Ni) substrate. Since the protective behavior of a passive film is influenced by its denseness, thickness, defects, and defect diffusivity^38^, it appears that the UV irradiation affects the formation of oxide films on the nanocrystalline Cu and Ni surfaces differently, leading to different variations in their properties. The oxide or passive films formed on different surfaces (after UV irradiation) play different roles of protection, which is in agreement with results of the polarization measurement.

It is clear that the UV light irradiation changes the surface properties through influencing the formation of oxide film, resulting in different trends of change in corrosion behaviors of the Cu and Ni foils. In order to understand the observed changes caused by the UV irradiation, surface performances and oxide films on the foils were further studied.

#### Electron work function

In order to obtain further information about the effect of UV irradiation on the corrosion behavior of the nanocrystalline metal foils, surface electron work functions (EWFs) of the foils were measured, including before and after exposure to the UV irradiation for 36 hours. EWF is related to the surface stability and can reflect the corrosion behavior of materials. Electron work functions of Ni and Cu foils with and without UV light irradiation were measured and obtained values are presented in Table 4. As illustrated, EWFs of both Ni and Cu after exposure to the 254 nm UV light (corresponding to the radiant energy of 4.84 eV) show rises, indicating that the UV light irradiation affects the stability of foil surfaces. This could be ascribed to the fact that the UV light activated the surfaces and rendered them easier to be oxidized and form thick oxidation films. Since the thickness of nature oxidation films on Cu and Ni surfaces is only a few nm or less than 10 nm^39,40^, the increased EWFs of Cu and Ni are possibly caused by the formation of thicker oxide films. Another possible factor is that the UV irradiation may influence the oxide type, leading to changes in work function. Although the UV light irradiation increased EWFs of both the Cu and Ni foils, the anti-corrosion property of Cu was improved but that of Ni was deteriorated. The opposite changes in EWF, along with results of corrosion tests, imply that the changes in anti-corrosion property of the metal foils caused by the UV irradiation should be more related to changes in oxide film’s completeness and properties.

**Table 4:** EWFs of the Cu and Ni measured before and after exposure to the UV illumination for 36 hours.

EWF(eV)	Cu	Ni
Before UV illumination	5.12	5.49
After UV illumination	5.32	5.62

We would like to indicate that the measured EWF value of a metal is influenced by its surface condition, e.g., oxide film, strain and roughness, etc. In the present study, we measured EWFs of a Cu or Ni foil before and after it was exposed to UV irradiation for a certain period without changing its other surface parameters. Thus, the measured changes in EWF should solely reflect the effect of UV irradiation on surface activity or condition of the metal.

#### Surface structure and properties

**Figure 5:**
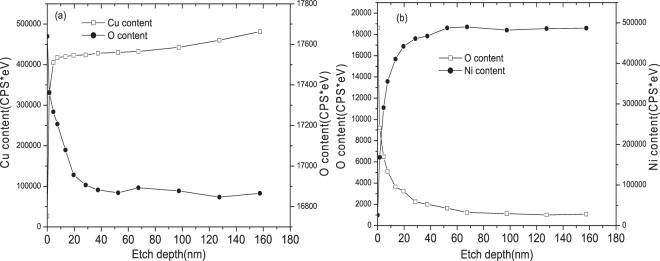
Cu and Ni concentrations vs. etching time (i.e. the duration of Ar ion sputtering), from which the thickness of oxidation film without UV irradiation can be determined: (**a**) Cu, (**b**) Ni.

**Figure 6:**
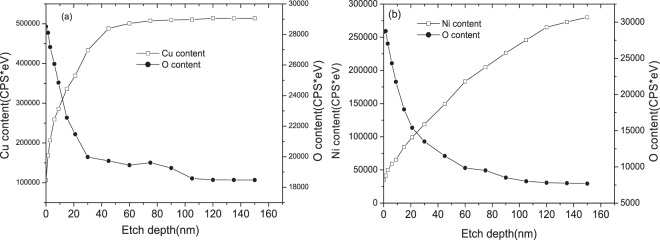
Cu and Ni concentrations vs. etching time, from which the thickness of oxidation film involving UV irradiation can be determined: (**a**) Cu, (**b**) Ni.

Using the method of XPS, the effect of UV irradiation on the depth or thickness of oxidation film on metal foils can be investigated. Concentrations of copper, nickel, and oxygen atoms in surface layers of the Cu and Ni foils without the UV irradiation versus the etching time (i.e. the duration of ion sputtering) are shown in Fig. 5(a,b), respectively. While the concentrations of copper, nickel, and oxygen atoms in surface layers of Cu and Ni foils after UV light irradiation versus the etching time are shown in Fig. 6(a,b), respectively. As illustrated, the XPS profiles acquired from Cu and Ni without UV light irradiation do not show large difference from those measured after the UV light irradiation.

In the figures, the etching time corresponds to the duration of sputtering by Ar ions. This means that the atomic concentration at zero etching time corresponds to the surface of the oxidation film. As the etching time increases, the concentrations of Ni and Cu increase while the concentration of O decreases. According to the literature^41^, the location where the oxygen concentration drops to 50% of its maximum value is defined as the boundary between oxide layer and matrix. It can be concluded that the thickness of the oxidation films without UV light irradiation were both less than 10 nm on Cu and Ni surfaces, respectively. While the thickness of the oxidation films after UV light irradiation was increased to 15 nm and 30 nm on Cu and Ni surfaces, respectively. The oxidation films are thicker than natural ones without UV light irradiation involved. Clearly the UV light irradiation plays an important role in affecting the metal surface oxidation, resulting in changes in the corrosion resistance of the metals.

**Figure 7:**
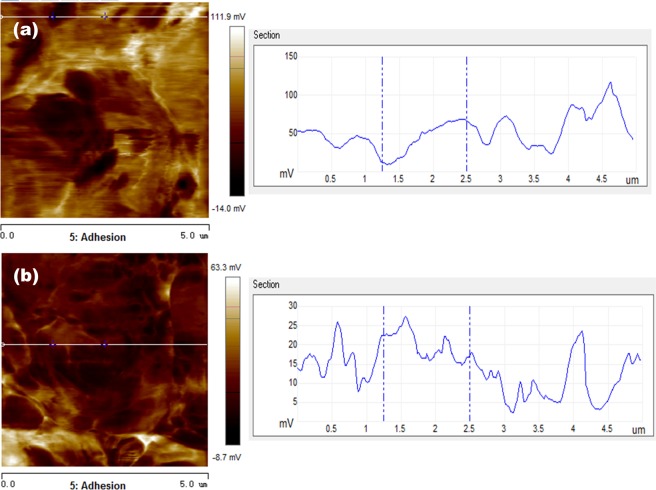
Adhesion distribution images and line profiles of the voltage of Cu foil in different irradiation conditions. (**a**) Adhesion distribution image before UV light irradiation (left-side), and the line profile of voltage (right-side) measured along the line shown in the adhesion distribution image; (**b**) adhesion image after UV light irradiation (left-side), and the line profile of voltage (right-side) measured along the line shown in the adhesion distribution image.

**Figure 8:**
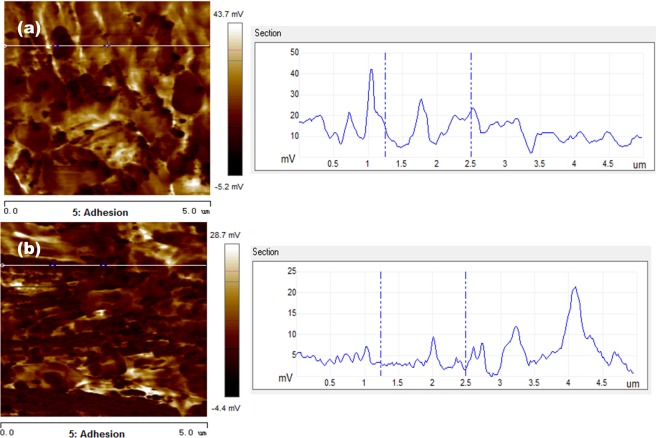
Adhesion distribution images and line profiles of the voltage of Ni foil in different irradiation conditions. (**a**) Adhesion distribution image before UV light irradiation (left-side), and the line profile of voltage (right-side) measured along the line shown in the adhesion distribution image; (**b**) adhesion image after UV light irradiation (left-side), and the line profile of voltage (right-side) measured along the line shown in the adhesion distribution image.

**Figure 9:**
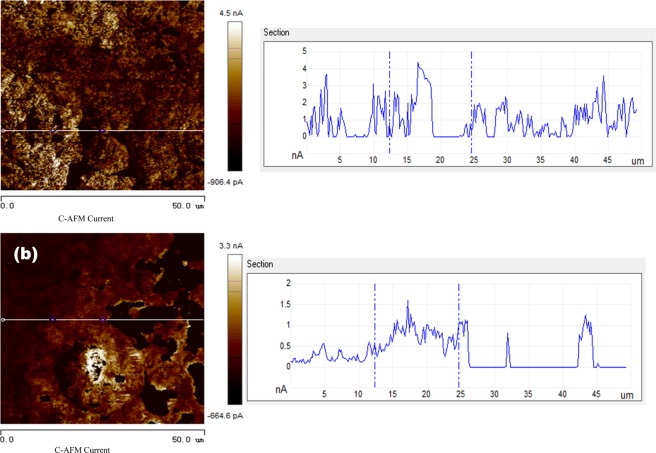
Current distribution maps and line profiles of the current of Cu foil at the different irradiation conditions. (**a**) Current distribution map before UV light irradiation (left-side), and the line profile of current (right-side) measured along the line shown in the current distribution map; (**b**) current distribution map after UV light irradiation (left-side), and the line profile of current (right-side) measured along the line shown in the current distribution map.

**Figure 10:**
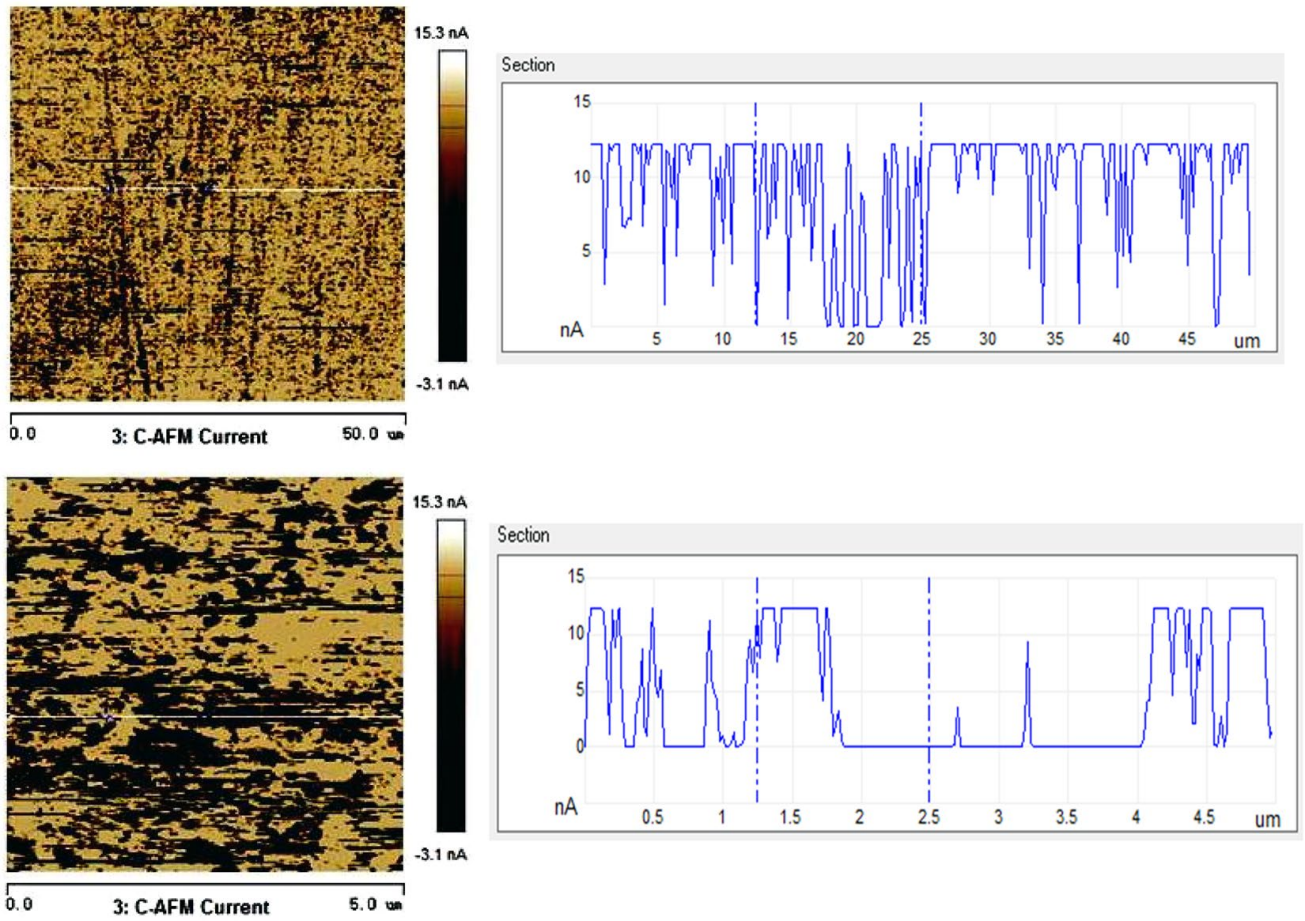
Current distribution maps and line profiles of the current of Ni foil at the different irradiation conditions. (**a**) Current distribution map before UV light irradiation (left-side), and the line profile of current (right-side) measured along the line shown in the current distribution map; (**b**) current distribution map after UV light irradiation (left-side), and the line profile of current (right-side) measured along the line shown in the current distribution map.

Tables 5 and 6 show adhesion behaviors and electrical currents of the metal foils before and after UV light irradiation, respectively. Corresponding adhesion and current distribution maps are illustrated in Figs. (7–10).

**Table 5:** Average adhesion values of the foils before and after UA irradiation.

Adhesion (mV/μN)	Cu	Ni
Before UV light	48.3/2.61	16.8/0.91
After UV light	17.7/0.96	8.05/0.44

**Table 6:** Average conductance values of the foils before and after UV irradiation.

Current (nA/)	Cu	Ni
Before UV light	0.929	9.85
After UV light	0.516	6.12

The presented value of adhesion is a measure of the interaction between the AFM probe and the metal surface or the adhesive force, which is calculated using the formula, F = U × k × f [F - adhesive force (N); U – voltage (V); k – the sensitivity coefficient (135*10^−9^ m/V); f - the spring constant of AFM probe (400 N/m)]. Since the adhesive force is proportional to the voltage, the voltage is also used to assess the adhesion behavior or the adhesive force. From Table 5, one may see that the adhesion values of both Cu and Ni foils decrease after exposure to the UV light irradiation. The decrease in adhesion of Cu (about 30 mV on average) is more than that of Ni foil (about 8 mV on average). Since the degree of adhesion reflects the surface activity, the above phenomena implies that the UV-modified oxide film on Cu is more stable with higher integrity than that on Ni.

The UV-induced increases in EWF and decreases in adhesion of the foils suggest that the UV irradiation resulted in changes in the type of oxide films on the metals. Although the thickening of oxide film as demonstrated by the XPS analysis could influence the surface work function, the influence is minor, since the oxide thickness should not affect much the adhesion behavior or the adhesive force.

In Table 6 and following Figs. 9 and 10, the presented values of current refer to the current flowing through the metal surface measured when bias voltage was applied to the AFM probe. In Table 6, one may see that the UV light irradiation more or less reduced the currents of both Cu and Ni foils, indicating that the formed oxide films on Cu and Ni obstruct electrons to pass through the oxide films, which are in line with the results of electron work function measurement. However, it should be pointed out that although the UV irradiation increased the apparent stability of surface oxide with higher EWF and lower current in the ambient condition, the formed oxide films are not necessarily strong when exposed to aggressive environments. As illustrated in Table 2 and Fig. 3, the UV light irradiation improved the anti-corrosion property of Cu but had a reversed influence on that of Ni when tested in the dilute NaCl solution.

Figures 9 and 10 illustrate distribution current maps and line current profiles of Cu and Ni foils before and after the UV light irradiation. Domains having different colors possess different values of electric current which reflects local conductance. The light color represents high conductance while the darker domains have lower conductance.

The current map image of the Cu foil (Fig. 9) before UV light irradiation shows light bright color with a smaller fraction of darker regions. While the current map of Cu foil after UV irradiation shows darker color with an increased fraction of darker regions. The peak current is higher before the UV irradiation, which is about 4 nA. After the UV irradiation, the current decreases to 1.5 nA. Besides, many dark areas show zero-current, implying that due to the UV light irradiation the oxide film has a larger coverage and is thicker than the oxide film formed in nature, more effectively obstructing electrons to pass through.

The Ni foil (Fig. 10) before UV light irradiation is brighter with less black spots than the Cu foil. After the UV irradiation, the Ni foil shows many dark areas. However, the peak current does not decline after the UV irradiation, though the current decreases to zero in the dark areas. This suggests that the UV irradiation increases the oxide coverage and thickness of the oxide film but may not change much its properties.

### Morphology and microstructure of corrosion products

**Figure 11:**
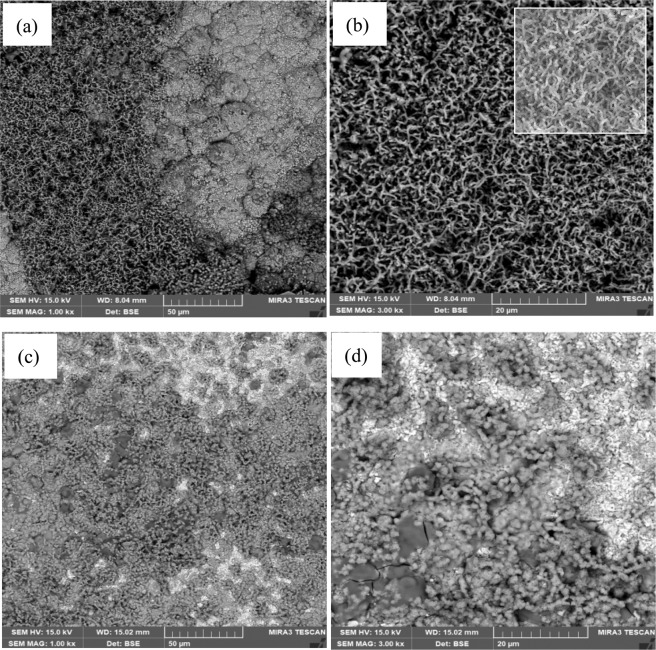
SEM of corrosion products on Cu foil with and without UV light irradiation. (**a,b**) SEM of corrosion products on Cu with UV light irradiation; a SE (secondary electron) image of the same area is inserted in (**b**). (**c,d**) SEM of corrosion products on Cu without UV light irradiation.

**Figure 12:**
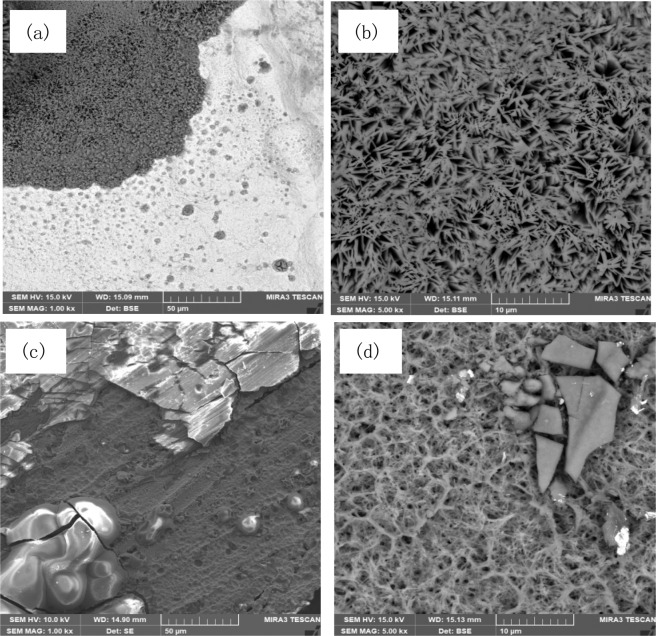
SEM of corrosion products on Ni foil with and without UV light irradiation. (**a,b**) SEM of corrosion products on Ni with UV light irradiation; (**c,d**) SEM of corrosion products on Ni without UV light irradiation.

For better understanding, we had a closer look at the corrosion products on the nanocrystalline Cu and Ni foils under SEM. Figures 11 and 12 show SEM surface images of Cu and Ni foils with and without UV light irradiation, taken after the foils experienced the immersion tests in the 3.5% NaCl solution.

The Cu foils, corroded under UV irradiation and in dark respectively, show very different surface morphologies. With the UV irradiation, the Cu surface displayed a homogeneous appearance, showing that compact and reticular long fiber structure (Fig. 11(a,b)), structures became more three-dimensional (3D) as corrosion proceeded. While the Cu surface without UV light irradiation (Fig. 11(c,d)) shows loose and short fiber structure. Corresponding EDS analysis indicates that both corrosion products formed with and without UV irradiation consist of CuCl_2_, CuCO_3_, Cu(OH)_2_ (data are not presented, since they are similar to those reported in the literature^42^). Cu-related solid compounds, including CuCl, Cu_2_O, CuO and Cu(OH)_2_, are formed during this process^42,43^. Based on the morphological observation and the EDS analysis, it can be inferred that the UV light irradiation favors the formation of continuous growth of reticular corrosion products with long oxide fibers on Cu surface. While without the UV light irradiation, corrosion products trend to form a reticular scale with short fibers. The reticular corrosion product with longer fibers formed on the Cu surface under the UV irradiation may reflect the oxide crystal growth with higher continuity. This could help reduce empty or discontinuous space in the corrosion product and thus decrease the penetration of corrosive medium. We would like to indicate that the corrosion product with longer fibers shown in Fig. 11(b) appears to have more empty space but this does not reflect the real situation. As shown by the SE image (secondary electron) of the same area inserted in Fig. 11(b), the corrosion product is rather compacted. Besides, long fibers are more beneficial to the fracture toughness, compared to short fibers. This could help improve the oxide film formed under the UA irradiation, e.g., enhancing its resistance to mechanical and electrochemical attacks^44^. As for the mechanism responsible for how the UV illumination favors the growth of long fibers, further studies are needed. It should be indicated that, though the structure factors of corrosion product, e.g., the fiber length, may influence its performance, the composition of the initial corrosion product plays a more crucial role in determining the corrosion process. This has been analyzed and discussed in the following section.

For the Ni foil, when corroded under UV irradiation and in dark respectively, their corroded surface morphologies are also different. The nickel corrosion product is in a form of fine and fiber network (Fig. 12(a,b)), and the structure shows more three-dimensional (3D) features when the corrosion proceeded in the absence of UV light irradiation. Under the UV irradiation, the structure of corrosion product became less homogeneous with large fragments (see Fig. 12(c,d)). The EDS results show that both corrosion products are mainly Ni(OH)_2_, NiCO_3_ and few NiCl_2_, which consistent with the literature^42^. As metallic Ni is exposed to neutral salt environments, a few soluble species such as chlorides, Ni(OH)^+^ and Ni(OH)_3_^−^ would be produced^42,45^. Besides, some fragments of solid compounds (poorly soluble phases under certain conditions) are formed on the metal surface, which could be an indicator of lower integrity of the corrosion product formed under the UV irradiation. Again, possible difference in composition between the corrosion products formed with and without the UV irradiation involved should be more responsible for the difference in their corrosion resistance. More detailed analysis on the compositional influence on the corrosion resistance of the oxide films formed with and without UV irradiation is presented and discussed in the following section.

### Characterization of oxide films

**Figure 13:**
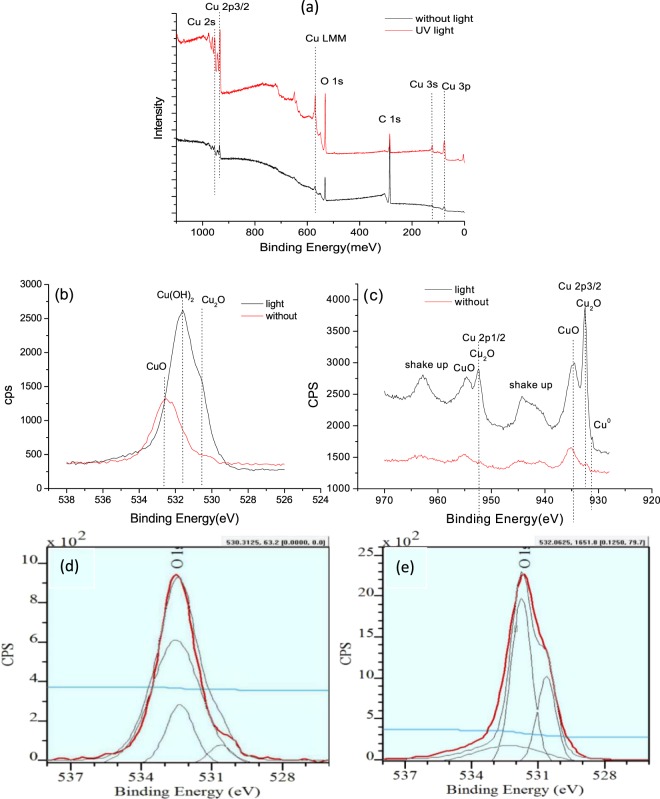
XPS spectra of surface products on the Cu foil. (**a**) Survey spectra of Cu foils with and without UV light irradiation; (**b**) O 1s, (**c**) Cu 2p 3/2, (**d**) detailed analysis for O 1s without UV light irradiation, and (**e**) detailed analysis for O 1s with UV light irradiation.

To obtain more information and understand the UV irradiation - induced changes in corrosion behavior, we conducted XPS analysis for the corrosion products formed with and without the UV light irradiation involved. Figure 13 depicts surface XPS spectra of the nanocrystalline Cu foil with and without UV light irradiation. Compared with the survey spectra, the sample after the UV light irradiation has stronger peaks in its XPS survey spectrum (see Fig. 13(a)). A high-resolution XPS of O1s peak centered at 532.6 eV is depicted in Fig. 13(a), confirming that the oxidation of Cu has been promoted under the UV irradiation. It should be mentioned that the existing carbon peak should come from some adventitious carbon contamination when samples have been exposed to the atmosphere.

Figure 13(b,c) display O 1s and Cu 2p3/2 high-resolution spectra, respectively. The peaks in Fig. 13(b) are centered at 530.67 eV, 531.6 eV and 532.46 eV. These peaks come from O^2−^ and OH^−^, and the corresponding products are Cu_2_O, Cu(OH)_2_ and CuO, respectively^42^. The ones at 931.6 eV, 932.46 eV and 934.44 eV in Fig. 13(c) respectively come from Cu^0^, Cu^+^ and Cu^2+^ of Cu 2p3/2. The strong peaks belong to Cu_2_O and CuO, respectively, which indicates that Cu is easy to be oxidized with or without UV light irradiation. The presence of shake-up peaks at 945 eV and 963 eV is ascribed to the formation of Cu_2_O^46–51^. The XPS results confirm that without the UV irradiation the main surface product on Cu is CuO and it considerably decreases after UV light irradiation. Through software analysis, it was determined that the fraction of CuO, Cu(OH)_2_ and Cu_2_O were 74.09%, 20.42% and 5.49%, respectively, before UV light irradiation, while the values changed to 20.21%, 53.67% and 26.31% after UV light irradiation. The above information demonstrates that the UV irradiation promotes the formation of Cu_2_O on Cu surface along with Cu(OH)_2_, while Cu is easier to form CuO under natural condition.

According to the outer layer electron configuration of Cu, the state of 3d^10^4s^1^ is easy to lose one electron and keep more stable state at higher energy level, e.g., at higher temperatures or under UV light irradiation. Since Cu_2_O is dense, well crystallized and more stable than CuO, it prevents Cl^−^ passing through and destroying the passive film and replacing O^2−^ to form copper chloride complexes. In combination with morphological analysis in section “Morphology and microstructure of corrosion products”, the longer fibers contain more Cu_2_O in the oxide film formed under UV irradiation, which is more protective against mechanical and electrochemical attacks. As a result, the corrosion resistance of Cu foil in 3.5% NaCl solution is improved after being illuminated by the UV light^13^. Meanwhile, studies showed that the oxide scale on Cu alloys containing appropriate amounts of Cu(OH)_2_ exhibited better anti-corrosion property^52,53^ in neutral environment. Thus, the increased amount of Cu(OH)_2_ in the oxide scale caused by the UV illumination also benefits the corrosion resistance of the Cu foil.

**Figure 14:**
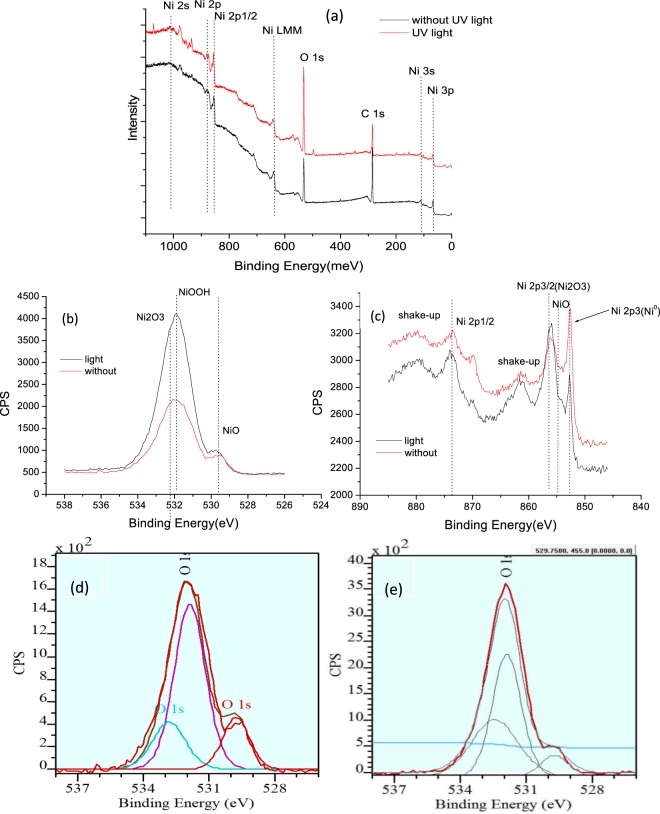
XPS spectra of surface products on the Ni foil: (**a**) Survey spectra of Ni foils with and without UV light irradiation; (**b**) O 1s, (**c**) Ni 2p 3/2, (**d**) detailed analysis for O 1s without UV light irradiation (**e**) detailed analysis for O 1s with UV light irradiation.

Figure 14 illustrates surface XPS spectra of the Ni foil with and without UV light irradiation. According to the survey spectra, the one with UV light irradiation displays a stronger O1s peak at 531 eV (see Fig. 14(a)), indicating that the UV light activates oxidation of Ni. Figure 14(b,c) exhibit O 1 s and Ni 2p3/2 high-resolution spectra respectively. The peaks at 529.8 eV, 532.1 eV and 532.6 eV shown in Fig. 14(b) originate from NiO, NiOOH and Ni_2_O_3_, respectively. The peaks at 852.4 eV, 853.7 eV and 856.6 eV^42,54^ in Fig. 14(c) match to Ni^0^, Ni^2+^ and Ni^3+^, respectively, and the strong peaks belong to Ni_2_O_3_ and NiO^55–60^, stating clearly that Ni is easy to be oxidized with or without UV light irradiation. Based on software analysis, it is determined that the fraction of Ni_2_O_3_, NiOOH and NiO were 19.97%, 54.81% and 25.22%, respectively, before UV light irradiation. While the values changed to 57.68%, 28.43% and 13.88% after UV light irradiation. The above XPS results identify that the surface product is a mixture of Ni_2_O_3_, NiO and NiOOH after the UV light irradiation, while without UV light irradiation the main products on Ni surface are NiO and NiOOH. From the obtained information, it can be concluded that Ni is much easier to form Ni_2_O_3_ under the UV light.

Ni_2_O_3_ is loose, porous, brittle, unstable, and easy to be destroyed by Cl^−^ ions, which can replace O^2−^ to form nickel chloride complexes^61^. Furthermore, it also increases the nucleation rate of pitting corrosion. While NiO is very stable and shows fully insulative property, and the electron cannot pass through it^45,62^. Usually the component of NiOOH is also chemically and thermally stability^63,64^ and can protect Ni from corrosion according to the literatures, which can prohibit the anion (Cl^−^) to pass through the oxidation film and corrode the substrate. Under the natural condition Ni is very easy to form dense and continuous NiO and NiOOH film, which has better anti-corrosion property. However, the Ni_2_O_3_ is more easily formed under ultraviolet irradiation, accompanied with sharp decreases in NiO. These may explain why the anti-corrosion property of Ni in 3.5% NaCl solution was deteriorated after being illuminated by UV light.

It is worth mentioning that due to the high-density grain boundaries in the nanocrystalline metal foils, which increase the surface activity and promote atom diffusion, the processes involved the above-mentioned reactions and oxide growth with and without the UV light irradiation should be accelerated when the substrate, either Cu or Ni, is in a nanocrystalline state.

## Conclusions

Influences of UV light irradiation on the corrosion behaviors of electrodeposited nanocrystalline Cu and Ni foils were studied. The presence of UV light irradiation in the Cl^−^ abundant environment reduced the corrosion rate of the Cu foil, but it showed an adverse effect on the corrosion rate of the Ni foil in the same environment. The corrosion rate of Cu foil was decreased by 70% after exposure to the UV light owing to the fact that the UV irradiation altered the ratio of various oxide components, resulting in the formation of a thicker, denser, continuous, more stable oxide film. However, the corrosion rate of Ni foil was increased over three-fold, and the anti-corrosion property of Ni foil was deteriorated by the UV light irradiation owing to the formation of a loose, porous, less stable oxide film on Ni surface after exposure to the UV light. This conclusions are corroborated by the obtained polarization curves, electrochemical impedance spectroscopy (EIS), and the XPS analysis.
